# Phosphatase kinetics reveal mechanistic pathways of soil phosphorus recovery during long-term desert revegetation

**DOI:** 10.1016/j.isci.2026.117614

**Published:** 2026-09-24

**Authors:** Mingzhu He, Chunming Xin, Chengyi Li, Huijun Qin, Jing Zhou, Shengzhong Du

**Affiliations:** 1 Key Laboratory of Ecological Safety and Sustainable Development in Arid Lands, Northwest Institute of Eco-Environment and Resources, Chinese Academy of Sciences, Lanzhou 730000, China; 2 Key Laboratory of Stress Physiology and Ecology in Cold and Arid Regions, Gansu Province, Northwest Institute of Eco-Environment and Resources, Chinese Academy of Sciences, Lanzhou 730000, P.R. China; 3 University of Chinese Academy of Sciences, Beijing 100049, China; 4 Documentation and Information Center, Northwest Institute of Eco-Environment and Resources, Chinese Academy of Sciences, Lanzhou 730000, P.R. China; 5 Key Laboratory for Information System of Mountainous Area and Protection of Ecological Environment of Guizhou Province, Guizhou Normal University, Guiyang 550001, China; 6 College of Resources and Environmental Sciences, Gansu Agricultural University, Lanzhou 730070, China

**Keywords:** phosphatase kinetics, phosphorus cycling, enzyme stoichiometry, soil biochemical recovery, dryland restoration

## Abstract

Phosphorus (P) limitation constrains dryland restoration, yet how phosphatase kinetics regulate soil P recovery remains unclear. We quantified P fractions and the activities and kinetic traits of alkaline phosphomonoesterase (PM), phosphodiesterase (PD), and inorganic pyrophosphatase (IP) across three desert restoration types: mobile dunes, a 31-year artificially revegetated desert, and natural vegetation in the Tengger Desert. Revegetation increased total, organic, and available P and enhanced PM and PD activities, substrate affinity, and catalytic efficiency, reflected by lower *K*_*m*_ and *K*_*m*_/*V*_*max*_. Multivariate analyses showed that phosphatase kinetic traits explained 67.9% of the variation in P fractions. PM and PD were mainly associated with organic P mineralization, whereas IP kinetics were more closely linked to Ca-bound P fractions. These results reveal complementary phosphatase pathways regulating P turnover during desert revegetation and identify enzyme kinetic traits as potential process-based indicators of biochemical recovery in dryland restoration.

## Highlights

• Revegetation boosts phosphatase activity, improving soil phosphorus availability
• Lower *K*_*m*_/*V*_*max*_ ratios in revegetated soils indicate higher enzyme efficiency
• Phosphatase kinetics explain 67.9% of variations in soil phosphorus fractions
• Enzyme kinetics insights support sustainable phosphorus management in deserts

## Graphical abstract

**Figure:**
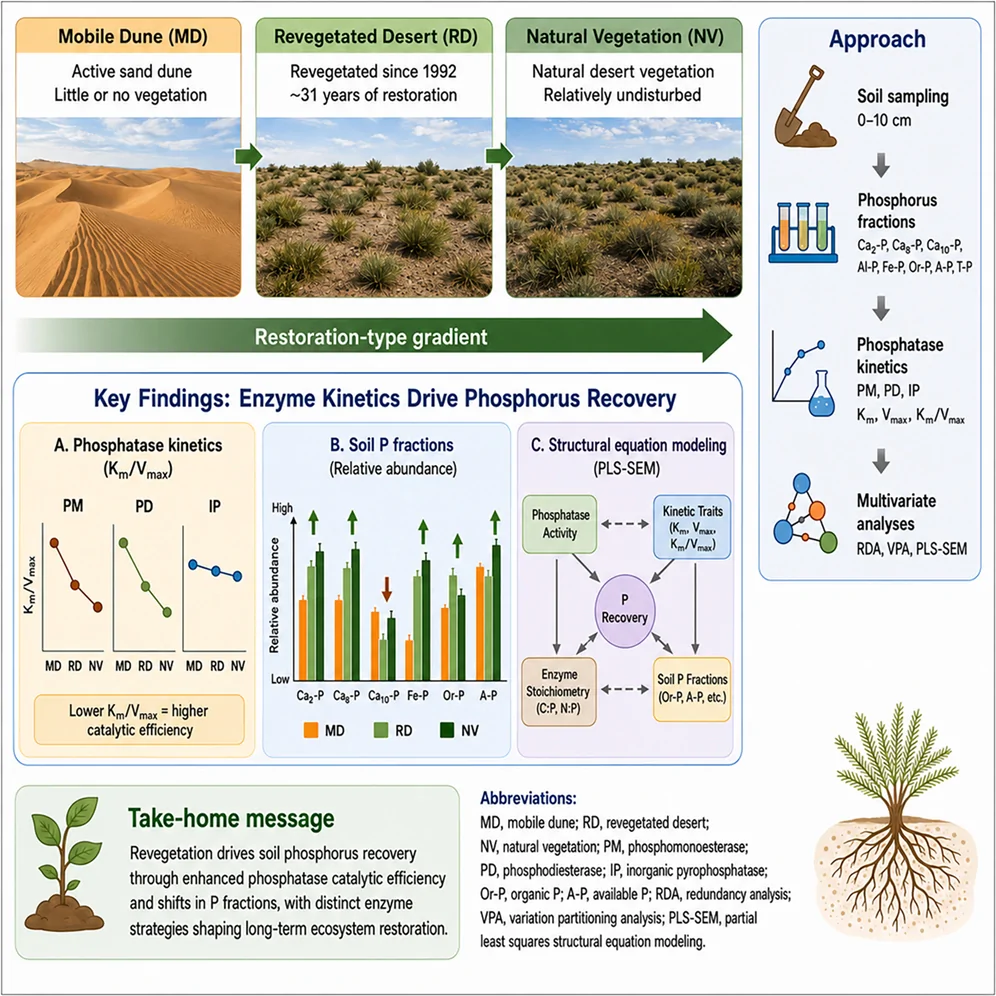

## Teaser

Soil science; Environmental science; Environmental chemistry; Soil chemistry

## Introduction

Phosphorus (P) is an essential nutrient for plant growth and microbial metabolism, with central roles in energy transfer, signal transduction, and the synthesis of nucleic acids and phospholipids.^1^^,^^2^ In arid and semiarid ecosystems, however, P availability is often constrained by sorption and precipitation with calcium, iron, and aluminum, which form mineral-associated P pools that are only partly accessible to plants and microorganisms.^3^^,^^4^^,^^5^ This limited P availability can restrict microbial activity, slow plant establishment, and constrain the recovery of soil fertility in degraded desert ecosystems. Understanding how soil P is transformed from relatively stable pools into biologically available forms is therefore critical for evaluating nutrient recovery and guiding ecological restoration in drylands.^6^^,^^7^^,^^8^

Revegetation is widely used to rehabilitate degraded desert ecosystems and can improve soil stability, organic matter accumulation, microbial activity, and nutrient retention.^9^^,^^10^ As plant communities develop, organic inputs from litter, roots, and rhizodeposition can alter both the quantity and composition of soil P pools. Organic P, available P, and calcium-associated inorganic P fractions, including dicalcium phosphate (Ca_2_-P) and octacalcium phosphate (Ca_8_-P), may therefore change substantially along restoration gradients.^5^^,^^11^ These changes are closely linked to soil phosphatases, which catalyze the mineralization of organic P compounds and participate in enzyme-mediated P turnover.^3^^,^^12^^,^^13^ Over longer restoration periods, vegetation development may not only increase enzyme activity but also reorganize the functional roles of different phosphatase types, thereby influencing the pathways through which P is retained, mineralized, or mobilized.^14^^,^^15^^,^^16^ However, the extent to which these enzyme-mediated processes regulate P recovery during long-term desert revegetation remains insufficiently resolved.

Soil phosphatases are key biochemical regulators of P cycling. Alkaline phosphomonoesterase (PM) hydrolyzes phosphomonoesters, alkaline phosphodiesterase (PD) acts on phosphodiester compounds such as nucleic acid- and phospholipid-derived substrates, and inorganic pyrophosphatase (IP) hydrolyzes inorganic pyrophosphate.^17^^,^^18^^,^^19^^,^^20^ These enzymes differ in substrate specificity and may therefore represent distinct pathways of P transformation. In alkaline desert soils, where calcium-associated P fractions are often abundant, phosphatase-mediated processes may be particularly important for linking biological P demand with the redistribution of organic and mineral-associated P pools.^4^^,^^15^^,^^21^ Yet most restoration studies have focused on phosphatase activity alone, whereas enzyme activity provides limited information on enzyme-substrate affinity, catalytic capacity, and functional efficiency.

Enzyme kinetic parameters offer a more mechanistic view of phosphatase function. Under the Michaelis-Menten framework, the maximum reaction velocity (*V*_*max*_) reflects catalytic capacity under substrate-saturated conditions, whereas the Michaelis constant (*K*_*m*_) reflects apparent substrate affinity. The ratio *K*_*m*_/*V*_*max*_ can be used as an inverse indicator of catalytic efficiency, with lower values indicating more efficient enzyme-substrate interactions under the assay conditions.^22^^,^^23^ These kinetic traits are sensitive to soil pH, organic matter inputs, substrate availability, microbial community composition, and environmental stress.^17^^,^^24^ They may therefore provide process-based information on how microbial and enzymatic systems respond to vegetation recovery. Nevertheless, few studies have integrated phosphatase kinetics with detailed P fractionation to identify the enzyme-specific pathways underlying soil P recovery in long-term desert revegetation.

To address this gap, we investigated how soil P cycling varies with phosphatase activity and kinetic traits across a desert restoration-type gradient comprising mobile dunes, revegetated desert, and natural vegetation (Figure 1). We focused on two mechanistic questions: (1) whether revegetation enhances the activities of PM, PD, and IP and (2) whether the kinetic traits of these enzymes, including *V*_*max*_, *K*_*m*_, and *K*_*m*_/*V*_*max*_, are linked to changes in soil P fractions. We hypothesized that long-term revegetation would enhance phosphatase activity and improve enzyme catalytic efficiency, reflected by lower *K*_*m*_ and *K*_*m*_/*V*_*max*_ values, thereby promoting organic P mineralization and the redistribution of available and calcium-associated P fractions. To test these hypotheses, we combined phosphatase activity and kinetic assays with sequential P fractionation, redundancy analysis, variation partitioning, and partial least squares structural equation modeling. This integrative approach allowed us to identify enzyme-specific pathways of P transformation and to evaluate phosphatase kinetic traits as potential process-based indicators of soil biochemical recovery during dryland restoration.

**Figure 1:**
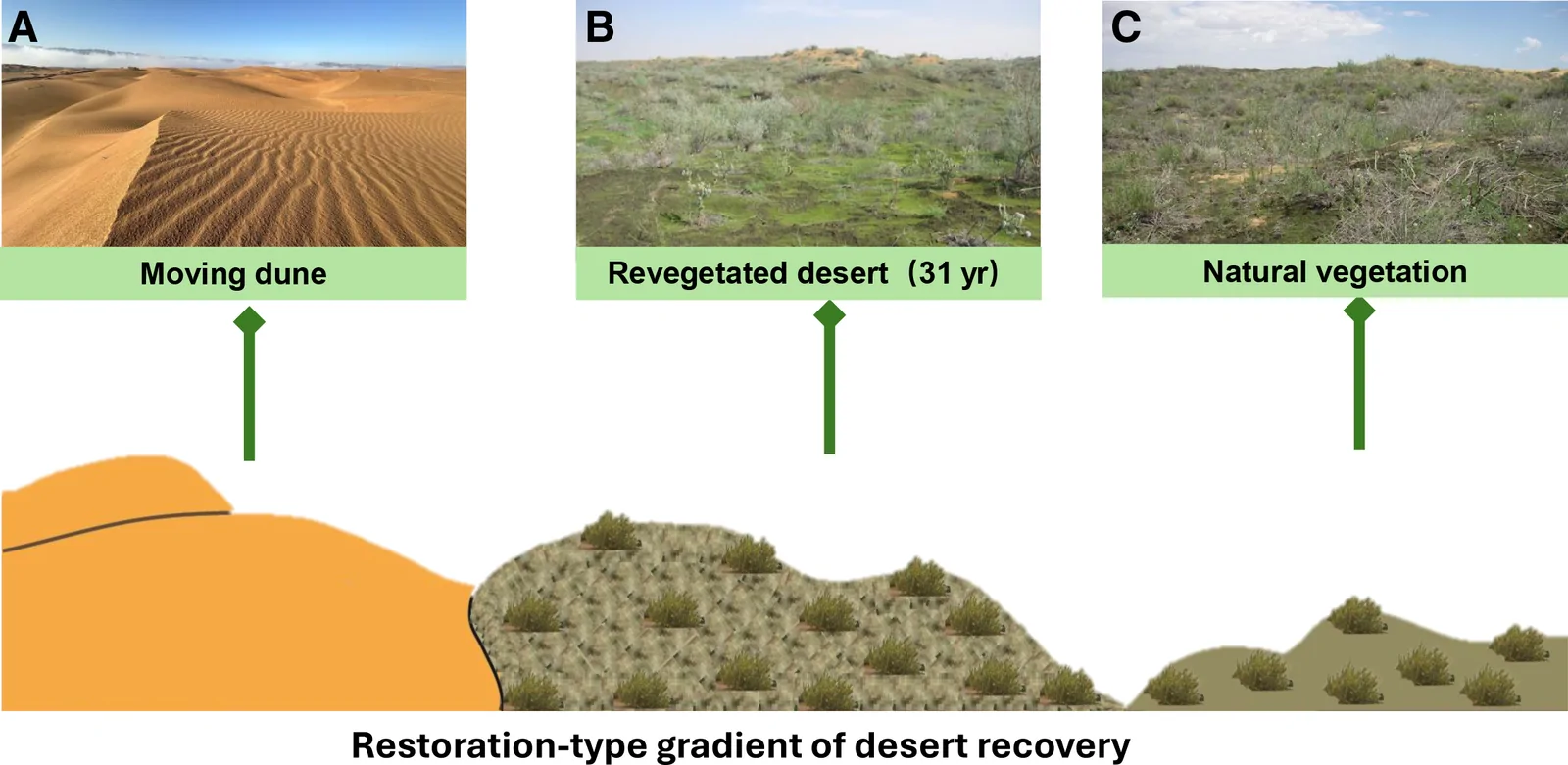
Study sites and restoration-type gradient in the Tengger Desert (A–C) Representative photographs and schematic illustration of the three restoration types used in this study: (A) mobile dune (MD), characterized by active sand movement, sparse vegetation, and minimal biological inputs; (B) revegetated desert (RD), established in 1992 and stabilized after approximately 31 years of revegetation, mainly with *Caragana korshinskii* and *Artemisia ordosica*; and (C) natural vegetation (NV), representing a relatively stable desert ecosystem with native plant communities and minimal direct anthropogenic disturbance. The lower schematic illustrates the spatial restoration-type gradient from mobile dune to revegetated desert and natural vegetation, which was used to assess changes in soil P fractions, phosphatase activity, and enzyme kinetic traits during long-term dryland restoration.

## Results

### Soil phosphorus fractions differ among desert restoration types

Soil P fractions differed markedly among the three desert restoration types: mobile dunes (MD), the 31-year revegetated desert (RD), and natural vegetation (NV). Compared with MD, RD showed significantly higher available P (A-P) and organic P (Or-P) (*p* < 0.05; Figure 2A), total P (T-P) and Ca_₁₀_-P (*p* < 0.05; Figure 2B), and Ca_₂_-P and Ca_₈_-P (*p* < 0.05; Figure 2C). These results indicate substantial P accumulation after long-term revegetation. Fe-P was significantly lower in RD than in MD (*p* < 0.05; Figure 2C), indicating that revegetation changed not only total P accumulation but also the distribution of P among mineral-associated fractions.

**Figure 2:**
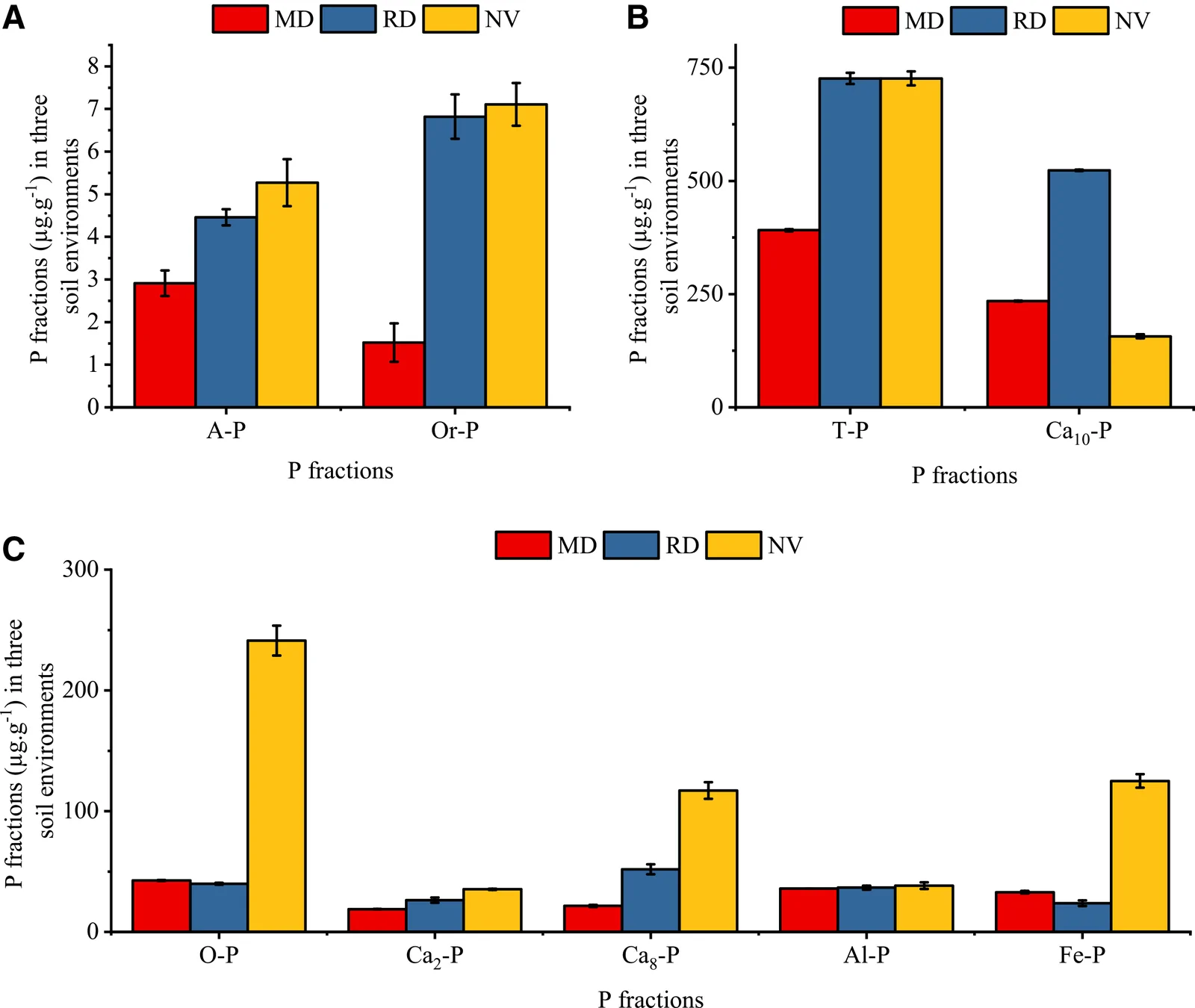
Changes in P fractions among three desert restoration types (A) A-P (available P) and Or-P (organic P): RD and NV showed significantly higher A-P and Or-P concentrations compared with MD. (B) T-P (total P) and Ca_10_-P (calcium-bound P): RD soils had higher T-P and Ca_10_-P levels than MD and NV. (C) O-P, Ca_2_-P, Ca_8_-P, Al-P, and Fe-P: NV soils had the highest levels of O-P, Ca_8_-P, and Fe-P, while MD showed the lowest concentrations across most fractions. Data are presented as mean ± SE (*n* = 3 biological replicates). The figure shows the concentrations of different P fractions in mobile dune (MD, red bars), revegetated desert (RD, blue bars), and natural vegetation (NV, yellow bars) soils. T-P, total phosphorus; A-P, available phosphorus; Or-P, organic phosphorus; Ca_2_-P, dicalcium phosphate; Ca_8_-P, octacalcium phosphate; Ca_10_-P, apatite phosphate; Fe-P, iron-bound phosphorus; Al-P, aluminum-bound phosphorus; O-P, occluded phosphorus.

Compared with NV, RD had similar T-P and Or-P concentrations but significantly lower A-P, Ca_2_-P, Ca_8_-P, and Fe-P concentrations (*p* < 0.05; Figures 2A–2C). In contrast, Ca_10_-P was significantly higher in RD than in NV (*p* < 0.05), indicating that a larger proportion of P remained in relatively stable calcium-associated forms in the revegetated soil. Overall, the 31-year artificially revegetated desert showed substantially improved soil P status relative to mobile dunes, but bioavailable and moderately labile P fractions had not fully converged with those in natural vegetation.

### Revegetation enhances PM and PD activity more strongly than IP activity

Phosphatase activities varied across the restoration-type gradient and substrate concentrations, showing enzyme-specific responses (Figure 3; Table 1). For alkaline phosphomonoesterase (PM) and phosphodiesterase (PD), activity increased with substrate concentration and reached the highest values at 125 mmol L^−1^. In RD, PM activity reached 0.723 μmol g^−1^ h^−1^, more than twice that in MD (0.297 μmol g^−1^ h^−1^) and close to the level observed in NV (0.717 μmol g^−1^ h^−1^) (Figure 3A). PD showed a similar pattern, with substantially higher activity in RD and NV than in MD (Figure 3B).

**Figure 3:**
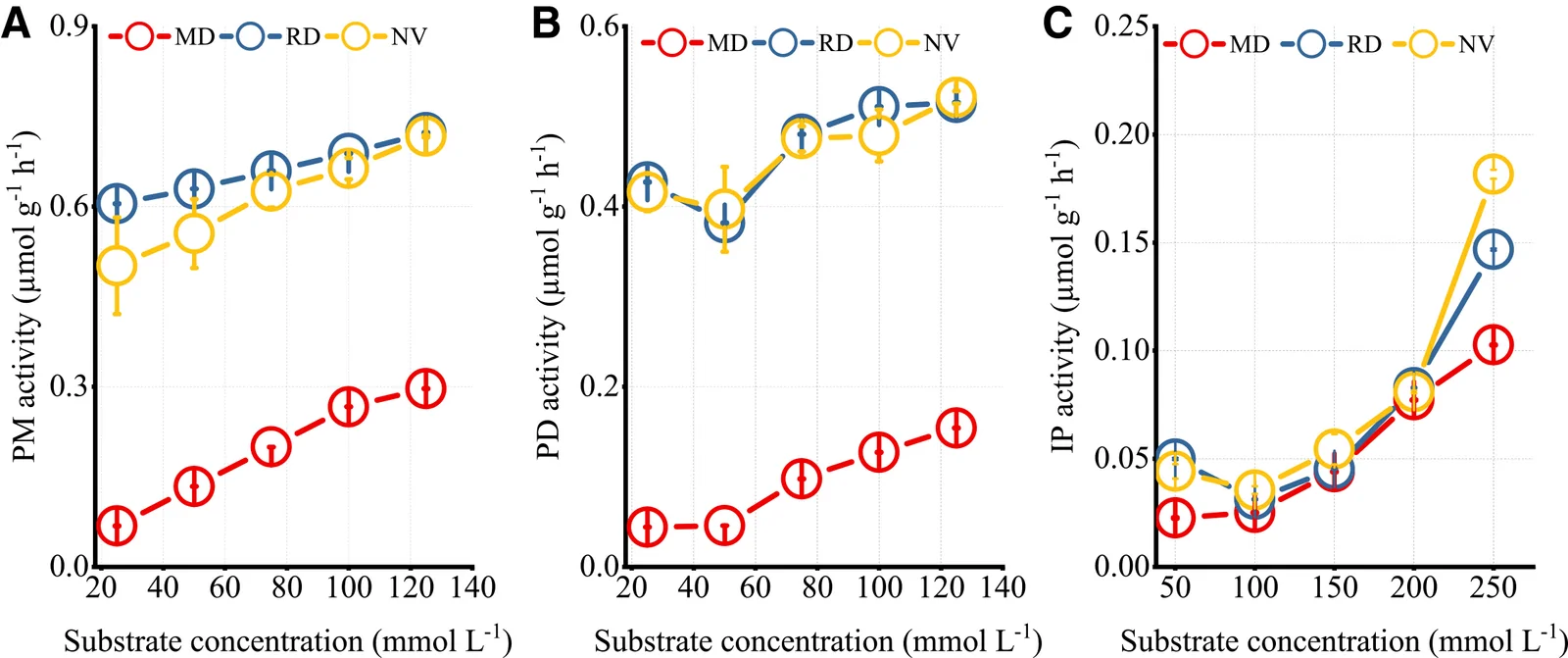
Changes in phosphatase activity with increasing substrate concentration among three desert restoration types (A) PM: RD and NV soils exhibited significantly higher PM activity compared with MD across all substrate concentrations. (B) PD: Similar trends were observed, with RD and NV showing higher PD activity than MD. (C) IP: NV soils displayed the highest IP activity at higher substrate concentrations, followed closely by RD, while MD consistently showed the lowest activity. This figure shows the enzymatic activity of three phosphatases under varying substrate concentrations in MD (red circles), RD (blue circles), and NV (yellow circles) soils. MD, moving dune; RD, revegetated desert; NV, natural vegetation; PM, alkaline phosphomonoesterase; PD, alkaline phosphodiesterase; IP, inorganic pyrophosphatase. Data are presented as mean ± SE (*n* = 3 biological replicates).

**Table 1:** Changes in phosphatase activities among three desert restoration types

Sites	PM (μmol.g^-1^ h^-1^)	PD (μmol.g^-1^ h^-1^)	IP (μmol.g^-1^ h^-1^)
	Substrate concentration (25–125 mmol L^−1^)	Substrate concentration (25–125 mmol L^−1^)	Substrate concentration (50–250 mmol L^−1^)
MD	0.068–0.297	0.044–0.154	0.023–0.103
RD	0.605–0.723	0.382–0.515	0.031–0.147
NV	0.502–0.717	0.397–0.521	0.036–0.182

**Two-way ANOVA**			

SC	*F* = 194.23, ∗∗∗*p* < 0.001	*F* = 115.39, ∗∗∗*p* < 0.001	*F* = 119.02, ∗∗∗*p* < 0.001
RT	*F* = 195.16, ∗∗∗*p* < 0.001	*F* = 164.01, ∗∗∗*p* < 0.001	ns

Note: This table shows the activity ranges of alkaline phosphomonoesterase (PM), alkaline phosphodiesterase (PD), and inorganic pyrophosphatase (IP) across three restoration types: mobile dunes (MD), revegetated desert (RD), and natural vegetation (NV). PM and PD were assayed at substrate concentrations of 25–125 mmol L^−1^, whereas IP was assayed at substrate concentrations of 50–250 mmol L^−1^. SC, substrate concentration; RT, restoration type; ns, not significant. Significance levels: ∗∗∗*p* < 0.001; ∗∗*p* < 0.01; ∗*p* < 0.05.

Inorganic pyrophosphatase (IP) activity increased more gradually with substrate concentration and reached the highest values at 250 mmol L^−1^ (Figure 3C). Across the restoration-type gradient, IP activity followed the order NV > RD > MD, but the effect of restoration type was weaker than that observed for PM and PD. ANOVA showed significant effects of substrate concentration on all three enzymes (*p* < 0.001) and significant effects of restoration type on PM and PD (*p* < 0.001), whereas the effect of restoration type on IP was not significant (*p* > 0.05; Table 1). These results indicate that long-term revegetation enhanced PM and PD activities more strongly than IP activity.

### Revegetation improves phosphatase substrate affinity and catalytic efficiency

Kinetic parameters further distinguished the responses of the three phosphatases across the restoration-type gradient (Figure 4; Tables 2 and S1). For PM, RD and NV showed much lower *K*_*m*_ and *K*_*m*_/*V*_*max*_ values than MD, indicating higher apparent substrate affinity and catalytic efficiency under the assay conditions. Although PM *V*_*max*_ was higher in MD than in RD and NV, this was accompanied by a much higher *K*_*m*_, resulting in lower overall catalytic efficiency in MD. For PD, RD and NV showed both higher *V*_*max*_ and lower *K*_*m*_ and *K*_*m*_/*V*_*max*_ than MD, indicating enhanced catalytic capacity and substrate affinity after revegetation.

**Figure 4:**
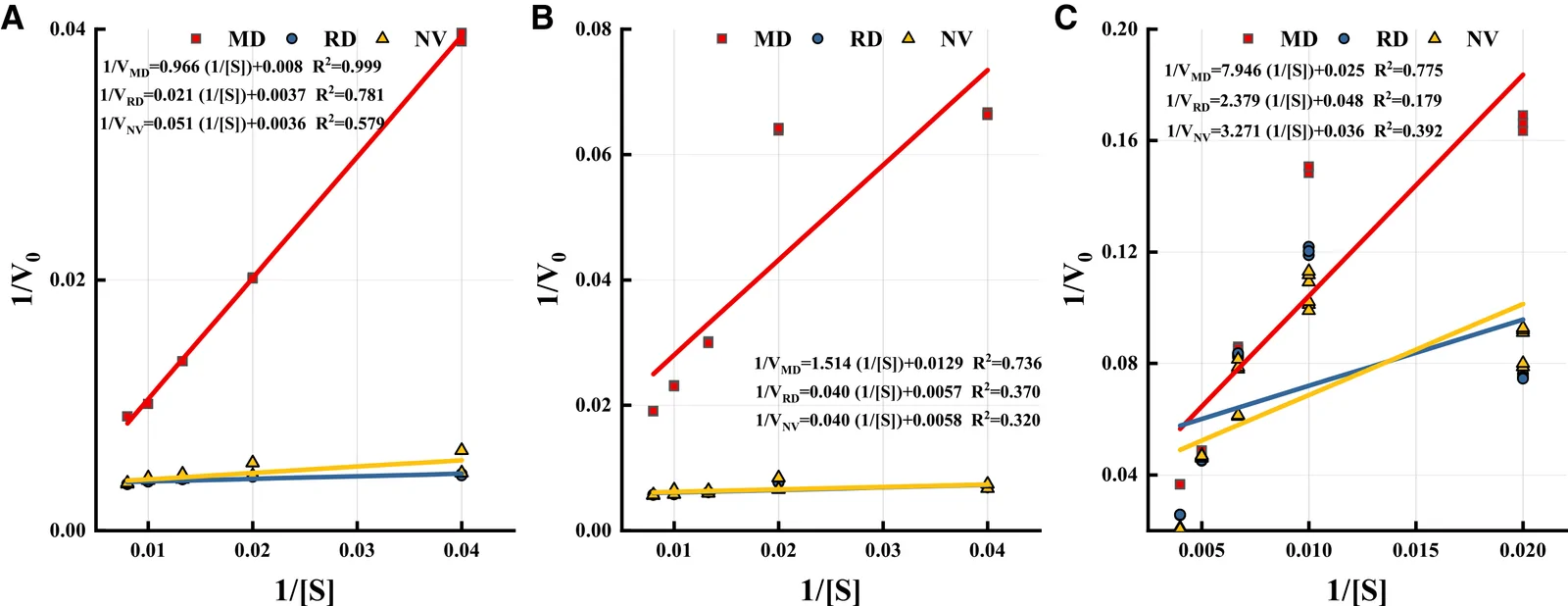
Lineweaver–Burk plots of phosphatase kinetics among three desert restoration types (A) Alkaline phosphomonoesterase (PM). (B) Alkaline phosphodiesterase (PD). (C) Inorganic pyrophosphatase (IP). The figure shows the double-reciprocal relationships between initial reaction rate (1/*V₀*) and substrate concentration (1/[S]) for mobile dune (MD, red squares), revegetated desert (RD, blue circles), and natural vegetation (NV, yellow triangles) soils. Linear regression equations and R² values are shown for each restoration type. MD, moving dune; RD, revegetated desert; NV, natural vegetation; PM, alkaline phosphomonoesterase; PD, alkaline phosphodiesterase; IP, inorganic pyrophosphatase.

**Table 2:** Changes in enzymatic kinetic parameters across three desert restoration types (mean ± SE)

	Sites	*K*_*m*_/*V*_*max*_	*V*_*max*_	*K*_*m*_
PM	MD	0.97 ± 0.01A	1226.19 ± 175.77A	1187.50 ± 184.56A
	RD	0.02 ± 0.00B	250.00 ± 0.00B	5.28 ± 0.92B
	NV	0.05 ± 0.03B	277.78 ± 39.28B	14.89 ± 9.39B
PD	MD	1.51 ± 0.01A	76.92 ± 0.00B	116.49 ± 0.43A
	RD	0.04 ± 0.00B	169.59 ± 4.14A	6.78 ± 0.17B
	NV	0.04 ± 0.01B	174.24 ± 7.58A	6.88 ± 0.94B
IP	MD	7.95 ± 0.15A	40.42 ± 1.31A	321.26 ± 16.54A
	RD	2.38 ± 0.05C	20.76 ± 0.18C	49.40 ± 1.36C
	NV	3.27 ± 0.57B	28.31 ± 3.72B	94.69 ± 28.33B

Note: This table presents the kinetic parameters (*K*_*m*_*/V*_*max*_, *V*_*max*_, and *K*_*m*_) for three phosphatases: alkaline phosphomonoesterase (PM), alkaline phosphodiesterase (PD), and inorganic pyrophosphatase (IP) in three restoration types: mobile dune (MD), revegetated desert (RD), and natural vegetation (NV). *K*_*m*_*/V*_*max*_, is an inverse index of apparent catalytic efficiency, with lower values indicating higher apparent catalytic efficiency; *V*_*max*_, denotes maximum reaction rate of the enzyme, and higher values suggest greater enzymatic activity potential; *K*_*m*_, represents Michaelis constant, representing the substrate concentration at which the reaction rate reaches half of *V*_*max*_, and lower *K*_*m*_ indicates higher substrate affinity. Statistical significance: Different uppercase letters (A, B, C) indicate statistically significant differences (*p <* 0.05) among the three restoration types for each parameter.

IP kinetics showed a different pattern. All three kinetic parameters of IP differed significantly among MD, RD, and NV (*p* < 0.05; Table 2). RD had lower *K*_*m*_ and *K*_*m*_/*V*_*max*_ than MD, indicating improved substrate affinity and apparent catalytic efficiency after revegetation. However, IP did not show the same convergence between RD and NV as observed for PM and PD. These results show that long-term revegetation promoted the recovery of phosphatase kinetic efficiency, particularly for PM and PD, whereas IP kinetics showed a more distinct pattern across restoration types.

### Phosphatase traits are linked to soil phosphorus fractionation

Redundancy analysis showed enzyme-specific associations between phosphatase traits and soil P fractions (Figure 5). PM and PD activities were positively associated with Or-P and Ca_8_-P and negatively associated with Al-P and A-P (Figure 5A). In contrast, IP activity was more closely associated with Ca_2_-P and A-P. These patterns indicate that different phosphatases were linked to different components of soil P fractionation across the restoration-type gradient.

**Figure 5:**
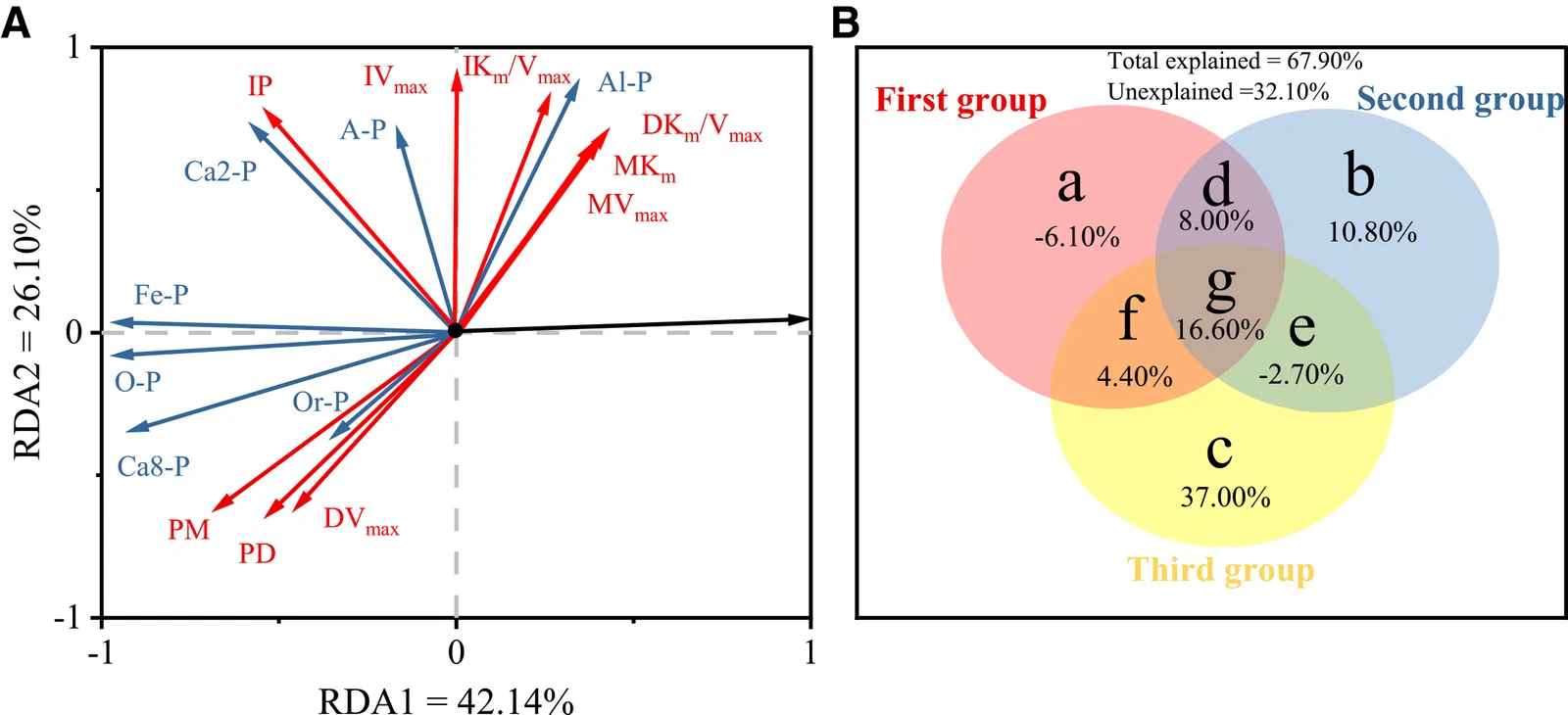
Effect of soil enzymatic kinetics on phosphorus fraction changes (A) Redundancy analysis (RDA): The plot shows the relationships between enzymatic parameters (*V*_*max*_, *K*_*m*_, *K*_*m*_/*V*_*max*_) and different P fractions (e.g., Ca_10_-P, Fe-P, Al-P, O-P). Arrows represent the direction and strength of these associations. (B) Variation partitioning analysis (VPA): The Venn diagram quantifies the contributions of enzymatic kinetics to P fraction changes. The three groups explain 67.9% of the variation, with 32.1% remaining unexplained. M*K*_*m*_ and M*V*_*max*_: *K*_*m*_ and *V*_*max*_ of alkaline phosphomonoesterase; D*K*_*m*_ and D*V*_*max*_: *K*_*m*_ and *V*_*max*_ of alkaline phosphodiesterase; I*K*_*m*_ and I*V*_*max*_: *K*_*m*_ and *V*_*max*_ of inorganic pyrophosphatase. This figure illustrates the relationship between enzymatic kinetics and phosphorus fraction changes in three desert soil environments.

The explanatory power of phosphatase activity also differed among enzyme types. PM activity explained 35.4% of the variation in P fractions (*p* < 0.01), followed by IP activity (31.6%, *p* < 0.05) and PD activity (28.3%, *p* < 0.05) (Table 3). Kinetic parameters also contributed significantly to variation in P fractions. For PM, *K*_*m*_ and *V*_*max*_ were associated with Al-P and A-P. For PD, *V*_*max*_ was closely aligned with Or-P and Ca_8_-P. For IP, *V*_*max*_ and *K*_*m*_/*V*_*max*_ were most strongly associated with Ca_2_-P (Figure 5B). Individual kinetic parameters explained 22.3%–25.4% of the variation in P fractions (Table 3).

**Table 3:** Explanatory power of enzymatic activity and kinetic parameters on phosphorus (P) fractions

Kinetic parameters	Explains%	*F*	*p*
PM	35.4	5.5	0.002
IP	31.6	4.6	0.01
PD	28.3	3.9	0.026
DK_m_/V_max_	25.4	3.4	0.022
DV_max_	24.5	3.2	0.02
MK_m_	24.4	3.2	0.026
IK_m_/V_max_	23.8	3.1	0.036
MV_max_	23.1	3	0.022
IV_max_	22.3	2.9	0.036

Note: This table presents the explanatory power (% Explains), *F* values, and *p* values of different enzymatic activities and their kinetic parameters in explaining variations in phosphorus (P) fractions across three desert soil environments. Explains %, proportion of variation in P fractions explained by each enzymatic parameter; *F,* F-statistic, indicating the strength of the relationship; *p*, statistical significance (*p* < 0.05 indicates significant relationships). Key parameters: PM, alkaline phosphomonoesterase; PD, alkaline phosphodiesterase; IP, inorganic pyrophosphatase, M*K*_*m*_ and M*V*_*max*_ represent the *K*_*m*_ and *V*_*max*_ of alkaline phosphomonoesterase; D*V*_*max*_ and D*K*_*m*_/*V*_*max*_ represent the *V*_*max*_ and *K*_*m*_/*V*_*max*_ of alkaline phosphodiesterase; I*V*_*max*_ and I*K*_*m*_/*V*_*max*_ represent the *V*_*max*_ and *K*_*m*_/*V*_*max*_ of inorganic pyrophosphatase.

Variation partitioning analysis further showed that phosphatase kinetic traits accounted for a large proportion of the variation in soil P fractions (Table S2). The kinetic trait sets associated with PM, PD, and IP explained 22.9%, 32.7%, and 55.3% of the variation, respectively, and their combined contribution reached 67.9% (*p* < 0.01). These results indicate that phosphatase kinetic traits were strongly linked to P fraction composition across the restoration-type gradient.

### Structural models identify enzyme-specific pathways of phosphorus recovery

Partial least squares structural equation modeling was used to evaluate the direct and indirect pathways linking phosphatase activity, kinetic traits, enzyme stoichiometry, and P fractions (Figure 6). Across the three enzyme-specific models, phosphatase activity affected P fractions mainly through pathways involving kinetic traits and stoichiometric variables rather than through direct pathways alone. In the PM and PD models, paths linking activity and kinetic-efficiency components were negative, whereas the IP model showed a positive association between activity and kinetic traits (Figures 6A–6C). These contrasting path structures indicate that PM, PD, and IP contribute to P recovery through different regulatory pathways.

**Figure 6:**
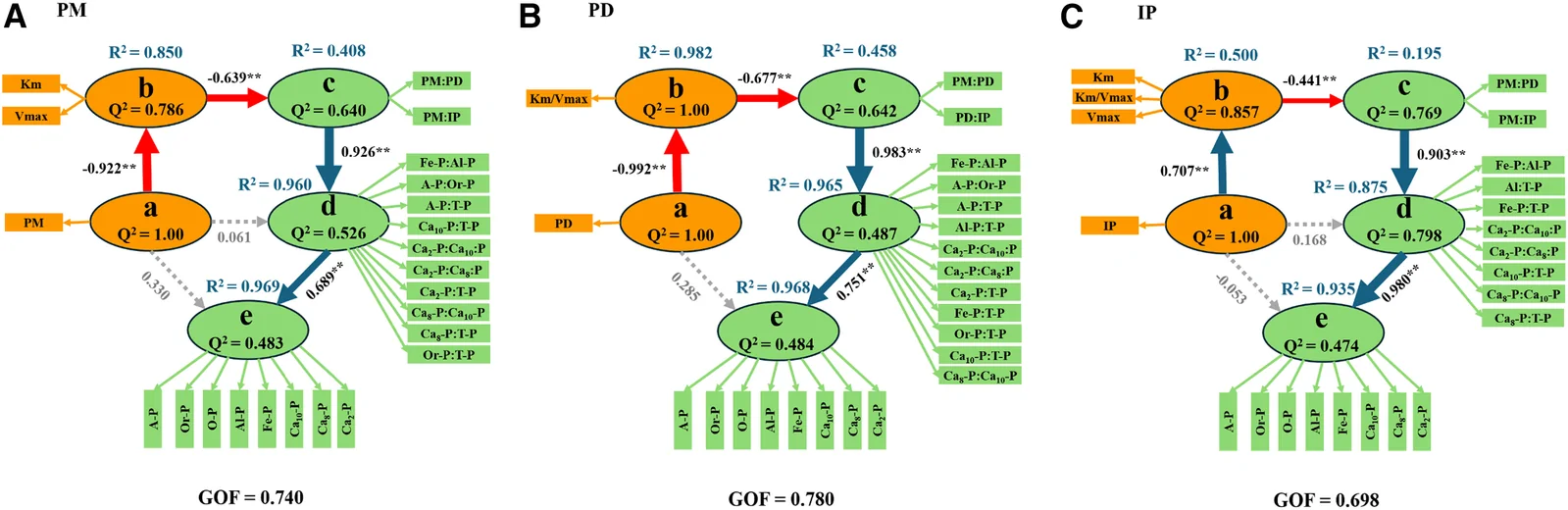
PLS-SEM analysis of phosphatase kinetics and their effects on phosphorus fractions (A) Alkaline phosphomonoesterase (PM): Shows pathways through which kinetic parameters (*K*_*m*_, *V*_*max*_) and enzymatic activity influence P fractions. (B) Alkaline phosphodiesterase (PD): Highlights the contributions of enzymatic kinetics and stoichiometry to P fraction changes. (C) Inorganic pyrophosphatase (IP): Displays the relationships between enzymatic efficiency and phosphorus fractions. This figure illustrates the partial least squares structural equation modeling (PLS-SEM) results, showing the relationships between enzymatic kinetics and phosphorus fractions for three phosphatases. Key symbols: a, Enzymatic activity; b, Kinetic process; c, Enzyme stoichiometry; d, P fraction stoichiometry; e, P fractions. Arrow colors: black arrows, positive correlations; red arrows, negative correlations. Model Fit: GOF (goodness-of-fit) values indicate strong explanatory power: PM: 0.740; PD: 0.780; IP: 0.698.

Together, these analyses show that long-term revegetation changed soil P fraction composition, enhanced phosphatase activity and kinetic efficiency, and strengthened the coupling between enzyme traits and P turnover. The results support an enzyme-specific framework in which PM and PD are more closely associated with organic P transformation, whereas IP is more closely linked to calcium-associated P fractions during dryland soil recovery.

## Discussion

### Revegetation promotes enzyme-mediated phosphorus recovery

Long-term revegetation reshaped soil P fractionation and phosphatase function across the restoration-type gradient. Compared with mobile dunes, the 31-year revegetated site had higher total P, organic P, available P, and several calcium-associated P fractions, indicating that vegetation establishment promoted P accumulation and redistribution in surface soils. This pattern is consistent with the gradual recovery of plant inputs, surface stabilization, microbial activity, and plant-soil feedbacks during dryland restoration.^9^^,^^10^^,^^21^ In mobile dunes, sparse vegetation and limited organic inputs constrain microbial colonization and extracellular enzyme production. As dune surfaces become stabilized, litter input, fine-root turnover, rhizodeposition, and the development of biological soil crusts in later successional types can create more favorable microsites for microbial activity and enzyme-mediated nutrient turnover.^11^^,^^25^^,^^26^

The responses of PM and PD suggest that organic P mineralization was particularly sensitive to revegetation. Both enzymes showed much higher activities in revegetated and natural vegetation soils than in mobile dune soils. Their lower apparent *K*_*m*_ and *K*_*m*_/*V*_*max*_ values indicate greater substrate affinity and catalytic efficiency under standardized assay conditions. These changes suggest that revegetation improved not only enzyme production but also enzyme-substrate coupling. In dryland soils, where low organic matter and limited water availability can constrain substrate diffusion and microbial activity, improved apparent kinetic efficiency may be important for sustaining P supply.^22^^,^^23^^,^^27^ The close associations of PM and PD with Or-P and Ca_8_-P further suggest that these enzymes are involved in the turnover of organic and moderately labile P pools during restoration.

The recovery of soil P cycling, however, remained incomplete after 31 years of revegetation. Although total P and organic P in the revegetated site were comparable to those under natural vegetation, available P, Ca_2_-P, Ca_8_-P, and Fe-P remained lower, whereas Ca_10_-P was higher. This pattern indicates that a considerable proportion of P in the revegetated soil was still retained in relatively stable mineral-associated forms. Thus, long-term revegetation improved soil P status relative to mobile dunes, but it did not fully re-establish the P availability pattern observed under natural vegetation. This incomplete convergence highlights the slow recovery of nutrient cycling in arid ecosystems and suggests that vegetation establishment alone may not be sufficient to restore all components of soil P turnover.

### IP kinetics reveal a distinct recovery pathway

A notable finding of this study is that IP kinetics were less responsive to revegetation than PM and PD kinetics. This muted response likely reflects the distinct substrate specificity and ecological role of IP. PM and PD act on broad pools of phosphomonoester and phosphodiester compounds derived from litter, roots, microbial residues, nucleic acids, and phospholipids. In contrast, IP specifically hydrolyzes inorganic pyrophosphate.^17^^,^^18^^,^^19^^,^^20^^,^^28^

This difference in substrate spectrum is important because the pyrophosphate pool is likely smaller, more transient, and more strongly regulated by mineral interactions than the more heterogeneous organic P pools targeted by PM and PD. As a result, increased organic matter inputs during revegetation may stimulate PM- and PD-mediated pathways more directly than IP-mediated pyrophosphate turnover.

Differences in microbial functional recovery may also contribute to the weaker IP response. PM and PD can be produced by a relatively broad range of microorganisms and plant roots, whereas IP-related processes may depend more strongly on specific microbial functions involved in pyrophosphate metabolism.^12^^,^^29^ Because microbial community composition and functional genes were not directly measured in this study, the responsible microbial groups cannot be identified here. Nevertheless, the kinetic pattern suggests that IP-related functions did not converge toward the natural vegetation condition as strongly as PM and PD did. This is consistent with our observation that PM and PD kinetic traits in the revegetated site were closer to those under natural vegetation, whereas IP followed a more distinct restoration trajectory.

Soil mineralogy may further constrain IP-related processes in alkaline desert soils. Calcium-associated P fractions are abundant in these soils, and pyrophosphate can interact strongly with mineral surfaces and calcium-rich phases.^30^ Such interactions may reduce substrate accessibility and weaken the apparent response of IP kinetics under standardized assay conditions. Therefore, the association between IP kinetic traits and Ca-bound P fractions should be interpreted as evidence of coupling between pyrophosphate turnover and calcium-associated P redistribution, rather than as direct enzymatic hydrolysis of all Ca-bound P pools. Together, substrate specificity, possible lags in microbial functional recovery, and mineral regulation of pyrophosphate accessibility provide plausible explanations for why IP kinetics were less responsive to revegetation than PM and PD.

### Phosphatase kinetics as process-based indicators of biochemical recovery

Phosphatase kinetic traits were closely associated with soil P fractionation across the restoration-type gradient. Variation partitioning showed that PM, PD, and IP kinetic traits together explained 67.9% of the variation in soil P fractions, indicating that enzyme kinetics captured a substantial part of the biochemical variation linked to P redistribution. Compared with enzyme activity alone, kinetic parameters provide additional information on catalytic capacity, substrate affinity, and apparent efficiency, and have been used to describe the functional potential of phosphatase-mediated P cycling.^23^ This distinction is important because high enzyme activity does not necessarily indicate efficient enzyme-substrate coupling, particularly when apparent *K*_*m*_ is also high. Kinetic traits can therefore help distinguish whether restoration mainly increases enzyme production, improves substrate affinity, or promotes both processes.

For restoration assessment, these parameters are best used as site-calibrated, process-based indicators rather than universal thresholds. Soil enzymes have long been used as biological indicators of soil quality because they are closely linked to nutrient cycling and respond sensitively to changes in soil management and ecosystem condition.^31^^,^^32^ Apparent *K*_*m*_, *V*_*max*_, and *K*_*m*_/*V*_*max*_ can be measured under standardized assay conditions and compared among restoration types, management treatments, and reference ecosystems. In this study, a favorable recovery pattern was reflected by the convergence of revegetated soils toward natural vegetation in key kinetic traits, especially lower apparent *K*_*m*_ and *K*_*m*_/*V*_*max*_ for PM and PD, together with increases in available P, organic P, soil organic matter, and vegetation cover.

Universal benchmark values for phosphatase kinetics should not be proposed at this stage because these parameters are influenced by soil type, pH, mineral composition, vegetation, microbial communities, and assay conditions. A more realistic approach is to establish local or regional reference ranges using natural vegetation, remnant stable ecosystems, or well-recovered restoration sites, consistent with reference-based approaches for evaluating soil functionality in restored semiarid ecosystems.^33^ In this framework, phosphatase kinetics can complement conventional chemical indicators such as total P and available P. Chemical indicators describe the size of P pools, whereas kinetic traits describe the biological capacity to transform and mobilize these pools. Combining these two types of information can provide a more complete assessment of soil biochemical recovery during dryland restoration.

### Implications for dryland restoration

The enzyme-specific responses observed here have implications for dryland restoration. The recovery of PM and PD suggests that revegetation can rebuild organic P mineralization pathways when plant inputs, microbial activity, and surface stabilization increase. Restoration strategies that enhance belowground carbon inputs and rhizosphere activity, such as the establishment of legumes or deep-rooted shrubs, may therefore favor enzyme-mediated P turnover.^9^^,^^34^ The more constrained response of IP, however, indicates that different P transformation pathways do not recover at the same pace. Monitoring programs should therefore consider multiple phosphatases rather than relying on a single enzyme indicator.

Future work should link enzyme kinetics with microbial community composition, P-cycling functional genes, and rhizosphere processes. Functional markers such as *phoD*, *phoA*, and genes involved in pyrophosphate metabolism could help determine whether changes in apparent kinetic traits are driven by microbial community shifts, enzyme production, or substrate accessibility.^35^ Measurements of root exudates and rhizosphere-specific enzyme kinetics would also clarify how plant traits regulate phosphatase recovery.^36^^,^^37^ Repeated monitoring across different dryland soil types will be needed to establish context-specific reference ranges for enzyme kinetic indicators and to test whether the patterns observed here are generalizable beyond the Tengger Desert.

Overall, this study shows that long-term revegetation promotes soil P recovery through enzyme-specific pathways. PM and PD were closely associated with organic P mineralization and showed clear recovery in activity and apparent catalytic efficiency, whereas IP followed a more constrained trajectory linked to calcium-associated P fractions. These findings indicate that soil P recovery after revegetation is not a single uniform process, but a set of complementary biochemical pathways that recover at different rates. Phosphatase kinetic traits therefore provide a mechanistic link between vegetation recovery, microbial biochemical function, and soil P availability, and may serve as potential process-based indicators of dryland soil restoration when calibrated against local reference conditions.

### Limitations of the study

This study used a restoration-type gradient rather than repeated measurements of the same plots through time. The three sites were selected within the same region and had broadly similar climatic and soil background conditions, making the sequence useful for comparing different restoration types of dune stabilization and vegetation recovery. Nevertheless, unmeasured site-level differences may have contributed to some of the observed patterns. The results should therefore be interpreted as restoration-associated differences, rather than a complete reconstruction of temporal recovery. Long-term monitoring of fixed plots would help separate directional recovery from residual spatial variation.

Our analyses focused on the 0–10 cm soil layer, where root activity, microbial processes, biological soil crust development, and P turnover are expected to be most active in desert soils. This design captured the most responsive part of the soil profile, but deeper layers may show different P fractionation patterns because of shrub rooting depth, soil water redistribution, and mineral-associated P storage. The enzyme kinetic assays were also conducted under standardized laboratory conditions. These assays are useful for comparing potential enzyme function among restoration types, but they cannot fully represent *in situ* constraints related to soil moisture, temperature, substrate diffusion, and microsite heterogeneity. Accordingly, *K*_*m*_, *V*_*max*_, and *K*_*m*_/*V*_*max*_ are interpreted as apparent kinetic traits under assay conditions rather than intrinsic constants of purified enzymes.

Microbial community composition, P-cycling functional genes, and root exudate profiles were not directly measured. These variables would help identify the biological drivers behind the observed kinetic patterns, particularly the weaker response of IP compared with PM and PD. Future studies combining enzyme kinetics with microbial functional markers, rhizosphere sampling, vertical soil profiles, and repeated field monitoring would provide a clearer understanding of how phosphatase-mediated P recovery develops during dryland restoration.

## Resource availability

### Lead contact

Requests for further information and resources should be directed to and will be fulfilled by the lead contact, Mingzhu He (hmzecology@lzb.ac.cn).

### Materials availability

This study did not generate new unique reagents, biological materials, or field materials.

### Data and code availability

• All data reported in this paper are provided in the main text, figures, tables, and supplemental information.
• This paper does not report original code.
• Any additional information required to reanalyze the data reported in this paper is available from the lead contact upon reasonable request.

## Acknowledgments

We sincerely thank the Scientific Editor and the two anonymous reviewers for their constructive comments and suggestions, which greatly improved the clarity and quality of the manuscript. This work was supported by the Excellent Doctoral Student Project of Gansu Province (no. 26JRRA163), the 10.13039/501100001809National Natural Science Foundation of China (grant no. 42577551), the “Light of West China” Program of the 10.13039/501100002367Chinese Academy of Sciences (grant no. E5220901), and the Key Program of the Natural Science Foundation of Gansu Province (no. 24JRRA081).

## Author contributions

M.H. conceived the study, developed the research framework, designed the experiment, supervised the project, interpreted the results, and led the writing and revision of the manuscript. C.X. contributed to experimental design, data interpretation, and manuscript revision. C.L. performed the data analysis, prepared the figures and tables, and contributed to the initial manuscript draft. H.Q., J.Z., and S.D. conducted the field sampling, laboratory analyses, and data collection. All authors discussed the results, reviewed the manuscript, and approved the final version.

## Declaration of interests

The authors declare no competing interests.

## STAR★Methods

### Key resources table

**Table:** 

REAGENT or RESOURCE	SOURCE	IDENTIFIER
**Chemicals, peptides, and recombinant proteins**		

Standard analytical reagents for soil P fractionation and phosphatase assays	Commercial sources	Standard laboratory grade
Substrates for PM, PD, and IP activity assays	Commercial sources	Standard enzyme assay reagents

**Software and algorithms**		

CANOCO 5	Microcomputer Power	CANOCO 5
Origin 2024	OriginLab	Origin 2024
Adobe Illustrator CS6	Adobe	Illustrator CS6
SmartPLS 4.1.0.3	SmartPLS GmbH	SmartPLS 4.1.0.3; https://www.smartpls.com

**Other**		

UV-visible spectrophotometer	Standard laboratory instrument	Used for colorimetric determination
Constant-temperature incubator	Standard laboratory instrument	Used for enzyme incubation
Soil auger	Standard field instrument	Used for soil core collection
2 mm soil sieve	Standard laboratory equipment	Used for soil sample preparation

### Experimental model and study participant details

Omitted because this study did not involve experimental model organisms, human participants, or animal subjects.

### Method details

#### Study area

This study was conducted at the Shapotou Desert Research and Experiment Station, Northwest Institute of Eco-Environment and Resources, Chinese Academy of Sciences, on the southeastern margin of the Tengger Desert, northern China (37°33′N, 105°02′E). The station is located at an elevation of 1,336.4 m above sea level. The region has a typical continental desert climate, characterized by cold winters, dry summers, frequent strong winds, and strongly seasonal precipitation. The mean annual temperature is 9.6°C, with recorded extremes ranging from −25.1°C to 38.1°C. Mean annual precipitation is 186.2 mm, approximately 80% of which falls between June and September.^34^

#### Restoration-type gradient and sampling design

To evaluate soil P dynamics and phosphatase function across different types of desert restoration, three representative sites were selected across the restoration-type gradient: mobile dunes (MD), revegetated desert (RD), and natural vegetation (NV) (Figure 1). The MD site consisted of active sand dunes with sparse vegetation and minimal biological inputs. The RD site had been stabilized through long-term revegetation initiated in 1992, primarily with *Caragana korshinskii* and *Artemisia ordosica*. Soil sampling was conducted in 2023, corresponding to approximately 31 years of vegetation recovery. The NV site was dominated by native plant communities and had experienced minimal direct anthropogenic disturbance.^4^^,^^34^

The three sites were located within the same desert region and shared broadly comparable climatic and soil background conditions. The MD site represented the early stage of dune stabilization, the RD site represented a long-term revegetated stage, and the NV site served as a reference condition with mature vegetation–soil interactions. This restoration-type gradient provided a field framework for comparing how long-term revegetation is associated with changes in soil P fractions, phosphatase activity, and enzyme kinetic traits.

#### Soil sampling and processing

Soil samples were collected between June and September 2023. At each site, three plots (3 m × 3 m each) were established. Within each plot, five soil cores were collected from the 0–10 cm layer using a hand auger and thoroughly mixed to form one plot-level composite sample. In total, nine biological composite soil samples were obtained across the restoration-type gradient, with three biological replicates per restoration type.

The 0–10 cm layer was selected because it represents the most biologically active part of desert soils, where root activity, microbial processes, biological soil crust development, and nutrient turnover are concentrated. After collection, samples were transported to the laboratory in cooled containers and divided into two subsamples. Fresh subsamples were gently passed through a 2 mm sieve to remove roots, stones, and coarse debris, and were stored at 4°C before phosphatase activity and kinetic assays. The remaining subsamples were air-dried at room temperature, sieved through a 2 mm mesh, homogenized, and used for soil P fractionation analyses.

#### Soil phosphorus fractionation

Total phosphorus (T-P) was determined by perchloric–sulfuric acid digestion. Available phosphorus (A-P) was extracted with 0.5 mol L^-1^ NaHCO_3_ at pH 8.0. Organic phosphorus (Or-P) was determined by ignition at 550°C followed by extraction with 0.2 mol L^-1^ H_2_SO_4_. Inorganic P fractions were measured using a sequential extraction procedure based on Zhang and Kovar^38^ and Bao.^39^ The extracted fractions included dicalcium phosphate (Ca_2_-P), octacalcium phosphate (Ca_8_-P), apatite phosphorus (Ca_10_-P), aluminum-bound phosphorus (Al-P), iron-bound phosphorus (Fe-P), and occluded phosphorus (O-P).

#### Phosphatase activity assays

Fresh soil subsamples stored at 4°C were used for phosphatase activity assays. The activities of alkaline phosphomonoesterase (PM), alkaline phosphodiesterase (PD), and inorganic pyrophosphatase (IP) were determined following established protocols.^19^^,^^20^ Assays were conducted under enzyme-specific buffer conditions, with pH 11.0 for PM and pH 8.0 for PD and IP. Soil suspensions were incubated with the corresponding substrates at 37°C, and product formation was quantified colorimetrically using a UV-visible spectrophotometer. For PM and PD, p-nitrophenol release was measured at 405 nm. For IP, the released inorganic phosphate was determined using the corresponding colorimetric procedure. Blanks were included to correct for non-enzymatic hydrolysis and background absorbance. Enzyme activities were calculated on an oven-dry soil mass basis and expressed as μmol g^-1^ h^-1^. The same assay conditions were applied to all samples to ensure comparability among restoration types.

#### Phosphatase kinetic assays

The kinetic characteristics of PM, PD, and IP were determined using substrate concentration gradients. Substrate concentrations were 25, 50, 75, 100, and 125 mmol L^-1^ for PM and PD, and 50, 100, 150, 200, and 250 mmol L^-1^ for IP. The initial reaction rate (*V*_0_) was calculated at each substrate concentration and expressed on an oven-dry soil mass basis.

Kinetic parameters were estimated within the Michaelis–Menten framework:

$$V_{0}=\frac{V_{\max}*\;\left[S\right]}{K_{m}+\left[S\right]}$$

where *V*_0_ is the initial reaction rate at substrate concentration [*S*], *V*max is the maximum reaction rate, and *K*_m_ is the Michaelis constant.

Apparent *V*_max_ and *K*_m_ values were estimated using the Lineweaver–Burk double-reciprocal transformation:

$$\frac{1}{V_{0}}=\left(\frac{K_{m}}{V_{\max}}\right)\left(\frac{1}{\left[S\right]}\right)+\frac{1}{V_{\max}}$$

For each enzyme and sample, 1/*V*_0_ was regressed against 1/[*S*], and apparent *V*_max_ and *K*_m_ were calculated from the intercept and slope of the fitted relationship. Because the Lineweaver–Burk transformation can disproportionately weight low-substrate observations, the resulting *K*_m_ and *V*_max_ values are interpreted here as apparent kinetic parameters under standardized assay conditions rather than intrinsic constants of purified enzymes. These parameters were used primarily for within-study comparisons among restoration types. The *K*_m_/*V*_max_ ratio was calculated as an inverse index of apparent catalytic efficiency, with lower values indicating more efficient enzyme–substrate coupling under the assay conditions.

### Quantification and statistical analysis

#### Kinetic parameter estimation

For each enzyme and soil sample, reaction rates were calculated across the corresponding substrate concentration gradient. Apparent *V*_max_ and *K*_m_ were estimated using the Lineweaver–Burk transformation, and *K*_m_/*V*_max_ was calculated as an inverse index of apparent catalytic efficiency. The same substrate gradients, incubation conditions, blanks, and analytical procedures were applied to all samples, allowing direct comparison among MD, RD, and NV soils. Technical replicates from laboratory assays were averaged before statistical analysis, and plot-level composite samples were treated as biological replicates.

#### Univariate statistical analysis

Data normality was assessed before parametric analysis. Differences in soil P fractions and phosphatase kinetic parameters among the three restoration types were tested using one-way analysis of variance (ANOVA), followed by Tukey’s HSD test. For phosphatase activity measured across substrate concentration gradients, the effects of restoration stage, substrate concentration, and their interaction were tested using two-way ANOVA where appropriate. Statistical significance was accepted at *p* < 0.05.

#### Multivariate analysis and structural modeling

Redundancy analysis (RDA) was used to evaluate associations between phosphatase traits and soil P fractions. Variation partitioning analysis (VPA) was used to quantify the relative and combined contributions of phosphatase kinetic trait groups to variation in soil P fractions. RDA and VPA were performed using CANOCO 5. Partial least squares structural equation modeling (PLS-SEM) was used to evaluate direct and indirect pathways linking phosphatase activity, enzyme kinetic traits, enzyme stoichiometry, and soil P fractions. PLS-SEM was performed using SmartPLS 4.1.0.3. The models were used to assess enzyme-specific pathways of P recovery across the restoration-type gradient.

#### Software

Univariate statistical analyses and figure generation were performed using Origin 2024. RDA and VPA were performed using CANOCO 5. PLS-SEM was conducted using SmartPLS 4.1.0.3. Final figure assembly was performed using Adobe Illustrator CS6.
