# Supplementary material for: Phosphatase kinetics reveal mechanistic pathways of soil phosphorus recovery during long-term desert revegetation
Source: iScience. 2026 Sep 24;29(10):117614. doi: 10.1016/j.isci.2026.117614 (PMC13635005; doi:10.1016/j.isci.2026.117614)
Supplement: Supplementary file 1 [file mmc1.pdf]

iScience, Volume *29*

Supplemental information

Phosphatase kinetics reveal mechanistic pathways of soil phosphorus recovery during long-term desert revegetation

Mingzhu He, Chunming Xin, Chengyi Li, Huijun Qin, Jing Zhou, and Shengzhong Du

Supplementary materials

Table S1. Slope of enzymatic activity variation across different substrate concentrations in three desert soil environments.

$k=\Delta E/\Delta S_b$	SC of PM and PD ($mmol.L^{-1}$)	PM			PD			SC of IP ($mmol.L^{-1}$)	IP		
		MD	RD	NV	MD	RD	NV		MD	RD	NV
k_1	25-50	0.97	0.37	0.79	0.02	-0.62	-0.26	50-100	0.01	-0.10	-0.05
k_2	50-75	0.98	0.44	1.05	0.71	1.34	1.06	100-150	0.10	0.07	0.10
k_3	75-100	0.99	0.43	0.55	0.40	0.41	0.05	150-200	0.18	0.20	0.14
k_4	100-125	0.45	0.52	0.80	0.37	0.06	0.57	200-150	0.14	0.34	0.54

Note: This table presents the slope values (*k*) representing the variation in enzymatic activity of alkaline phosphomonoesterase (PM), alkaline phosphodiesterase (PD), and inorganic pyrophosphatase (IP) across different substrate concentration ranges in Moving Dune (MD), Revegetated Desert (RD), and Natural Vegetation (NV) soils. Positive (+) values indicate an increase in enzymatic activity with increasing substrate concentration; Negative (–) values indicate a decrease in enzymatic activity with increasing substrate concentration. Abbreviations: SC: Substrate concentration.

Table S2. Results of variation partitioning analysis (VPA) for phosphorus (P) fractions.

Tested fraction	F	p
a+b+c+d+e+f+g	4.9	0.002
a+b+d+e+f+g	2.2	0.036
a+c+d+e+f+g	4.7	0.002
b+c+d+e+f+g	8.8	0.002
a+d+f+g	2.6	0.026
b+d+e+g	3.7	0.018
c+e+f+g	7.8	0.002

Note: This table presents the *F*-values and *p*-values for different tested fractions of variation partitioning analysis, illustrating the contributions of multiple factors to the variation in P fractions across three restoration types. Tested fractions, represent different combinations of explanatory variables (a, b, c, d, e, f, g) contributing to the observed variation. *F, F*-statistic indicating the strength of each fraction's contribution to variation; *p,* statistical significance ($p < 0.05$ indicates significant contributions). The variables (a, b, c, d, e, f, g) correspond to specific environmental and enzymatic factors, which are defined in the main text or related figures.
